# Effects of the monoamine stabilizer (-)OSU6162 on cognitive function in alcohol dependence

**DOI:** 10.1007/s00213-019-05345-6

**Published:** 2019-10-18

**Authors:** Lotfi Khemiri, Pia Steensland, Joar Guterstam, Örjan de Manzano, Johan Franck, Nitya Jayaram-Lindström

**Affiliations:** 1 Department of Clinical Neuroscience, Centre for Psychiatry Research, Karolinska Institutet & Stockholm Health Care Services, Stockholm County Council, Stockholm, Sweden; 2 Department of Neuroscience, Karolinska Institutet, Stockholm, Sweden

**Keywords:** Alcohol use disorder, Dopamine, Monoamine stabilizer, Impulsivity, Cognition, OSU6162

## Abstract

**Introduction:**

Alcohol dependence (AD) is associated with a dysregulated mesolimbocortical dopamine system—a pathway which is also implicated in both reward and cognition. The monoamine stabilizer (-)-OSU6162 (OSU) is a novel pharmacological compound with the ability to reduce ethanol intake and ethanol seeking in long-term drinking rats as well as reducing alcohol craving in AD patients. Dopaminergic drugs can both impair and improve cognitive functions, and the aim of the current study was to investigate the effect of OSU treatment on cognitive functioning in AD patients.

**Method:**

In a randomized double-blind placebo-controlled study, 56 individuals with AD received 14 days of OSU or placebo treatment. Neuropsychological tasks from the Cambridge Automated Neuropsychological Test Battery (CANTAB®) and other tasks were used to evaluate treatment effect on executive function/impulsivity, working memory, attention, emotional recognition, and divergent thinking.

**Results:**

Treatment with OSU did not impair neuropsychological function in any of the cognitive domains investigated (all *p* > 0.1). In fact, OSU treatment did, compared to placebo, improve future planning ability (*F*_(1,46)_ = 6.9; *p* = 0.012; Cohen’s *d* = 0.54), verbal divergent thinking (*F*_(1,44)_ = 10.1; *p* = 0.003; *d* = 0.96), and response time for emotional recognition (*F*_(1,47)_ = 6.7; *p* = 0.013; *d* = 0.44).

**Conclusion:**

OSU treatment did not cause short-term cognitive side effects, further supporting the potential of OSU as a clinically feasible pharmacological treatment in AD patients. OSU treatment might improve future planning, verbal divergent thinking, and emotional recognition latency, which in turn may have a beneficial impact on alcohol use outcomes. Future studies are needed to confirm these preliminary findings.

**Electronic supplementary material:**

The online version of this article (10.1007/s00213-019-05345-6) contains supplementary material, which is available to authorized users.

## Introduction

Alcohol dependence (AD) is a psychiatric disorder characterized by clinical symptoms such as loss of control, craving, an inability to quit drinking, and continued use despite negative consequences (American Psychiatric Association 2000). Although not explicitly stated in the diagnostic criteria, it is well established that AD is also associated with a wide array of cognitive deficits (Stavro et al. 2013; Le Berre et al. 2017). In a recent meta-analysis, it was found that AD patients exhibit significant cognitive impairments across all measured domains, including executive function, inhibition/impulsivity, and working memory (WM) (Stavro et al. 2013). Furthermore, previous studies have shown that cognitive outcomes at baseline, e.g., elevated self-rated impulsivity (Müller et al. 2008), risk-taking (Bowden-Jones et al. 2005), reduced WM (Teichner et al. 2001), and impaired response inhibition (Rupp et al. 2016), can predict relapse to drinking, in AD patients in clinical treatment settings. It has therefore been suggested that cognitive deficits could constitute a possible therapeutic target in the development of novel treatments for alcohol use disorders (Naqvi and Morgenstern 2015).

The syndrome of AD has been characterized as a reward deficiency syndrome (Koob 2013), and is associated with altered dopaminergic neurotransmission manifested as decreased dopamine receptor availability and dopamine release in the striatum (Volkow et al. 1996, 2007; Heinz et al. 2004; Martinez et al. 2005), as well as decreased dopamine transmission in the prefrontal cortex (Narendran et al. 2014). Dopamine acts by modulating fronto-striatal brain circuits (Arnsten and Li 2005; Robbins and Arnsten 2009) and is an important neurotransmitter not only in reward (Schultz 2002), but also for higher-order cognitive functions and decision-making (Rogers 2011). In fact, several pharmacological agents targeting dopaminergic neurotransmission have been shown to affect cognitive functions such as those related to impulse control (reviewed by (Dalley and Roiser 2012). For instance, the psychostimulant amphetamine, which increases synaptic dopamine (Koob and Bloom 1988), has been found to improve response inhibition and attenuate delay discounting in healthy volunteers (de Wit et al. 2002). Methylphenidate, an amphetamine derivate used as treatment of ADHD, is known to mediate its actions by blocking uptake of dopamine and noradrenaline (Berridge et al. 2006), and has been shown to also improve cognitive deficits such as risk-taking (DeVito et al. 2008) and response disinhibition (DeVito et al. 2009) in patients with ADHD. Furthermore, the stimulant modafinil, thought to act in part by inhibiting dopamine uptake (Mereu et al. 2013), has been shown to improve several cognitive domains, e.g., memory, planning, and response inhibition in both healthy volunteers (Turner et al. 2003) and in ADHD patients (Turner et al. 2004). In addition, modafinil has been shown to affect stop signal task performance (Schmaal et al. 2013) and WM in AD patients (Joos et al. 2013a) moderated by baseline performance, suggestive of a positive treatment effect only in patients with poorer performance on baseline cognitive tests. Finally, the partial dopamine agonist aripiprazole has shown some promise as a novel AD medication (Voronin et al. 2008; Myrick et al. 2010), and has also been suggested to improve cognitive function in schizophrenia patients (Bervoets et al. 2012; Shin et al. 2018; Veselinović et al. 2019). Taken together, dopaminergic neurotransmission plays an important role in the pathophysiology of AD and constitutes a potential pharmacological target for treating cognitive deficits.

The monoamine stabilizer OSU6162 (OSU) is a pharmacological compound developed by Arvid Carlsson and colleagues. The compound has the ability to enhance, inhibit, or have no effect on dopaminergic neurotransmission depending on the prevailing dopaminergic tone (Sonesson et al. 1994; Carlsson et al. 2004). The complete mechanism of action is not fully known; however, it has been suggested that OSU can act as an antagonist at both the pre-synaptic autoreceptors and postsynaptic D2 receptors, and thereby induce functionally opposite effects (Sonesson et al. 1994; Carlsson et al. 2004; Lahti et al. 2007; Rung et al. 2008). Furthermore, OSU has been suggested to act as a partial agonist on the serotonin 5-HT2A receptor (Burstein et al. 2011; Carlsson et al. 2011) and has high affinity for the sigma receptor (Sahlholm et al. 2013). OSU has been evaluated in healthy individuals (Rodríguez et al. 2004) as well as in patients with mental fatigue following stroke or traumatic brain injury (Johansson et al. 2012), Huntington’s disease (Kloberg et al. 2014), and chronic fatigue syndrome (Nilsson et al. 2018), and has been found clinically safe with a mild unspecific side effect profile (e.g., headache, gastrointestinal symptoms, fatigue).

Steensland and colleagues recently identified OSU as a potential treatment for AD by showing that the compound attenuates alcohol-mediated behaviors (Steensland et al. 2012) and restores striatal dopaminergic deficits (Feltmann et al. 2016) in alcohol-drinking rats. Importantly, OSU6162 did not induce condition place preference in neither alcohol-naïve nor long-term drinking rats, suggesting that the compound does not have reinforcing properties in itself (Feltmann et al. 2016). Based on these preclinical findings, we conducted the first randomized placebo-controlled laboratory study in AD patients, where OSU was found to attenuate craving and liking for alcohol after consumption of an alcoholic drink, with the greatest treatment effect in patients with high baseline impulsivity (Khemiri et al. 2015). More recently, we have demonstrated that OSU treatment improves motor impulsive behavior on the five-choice serial reaction time task in alcohol-naïve and long-term drinking rats (Fredriksson et al. 2018). Collectively, these studies have suggested that OSU is a promising novel pharmacotherapy candidate in AD, but to the best of our knowledge, the effect of OSU on cognitive function has not yet been evaluated in any clinical setting.

In addition to the potential of improving cognition, it is also known that pharmacological agents targeting the dopaminergic pathways can impair cognitive functions. For instance, dopamine agonists when administered to patients with Parkinson’s disease reduce impulse control, which can result in development of impulse control disorders (O’Sullivan et al. 2009; Corvol et al. 2018). It is therefore of great importance to assess whether novel dopaminergic therapeutics such as OSU are associated with impairments in cognitive function, as this could be detrimental for the desired clinical outcome and reduce compliance to treatment. The aim of the current study was thus to investigate the effects of 14 days of OSU treatment in AD patients on a wide array of cognitive functions, including executive function, impulsivity, attention, WM, emotional recognition, and divergent thinking.

## Methods

### Participants

The data reported in the current study was collected as part of a previously reported study investigating the effects of OSU on craving (Khemiri et al. 2015). In brief, we recruited 56 AD patients using public advertisements online and in local newspapers. All participants provided written informed consent before study participation and the study was approved by the regional ethical review board in Stockholm. The Swedish Medical Products Agency approved the study, but since this was the first study in AD patients, the treatment duration was restricted to 14 days. The study was registered in the European Clinical Trials Database (EudraCT; Identification number 2011-003133-34), and external monitoring was performed by the Karolinska Trial Alliance in order to guarantee that the study was conducted in accordance with the Declaration of Helsinki and Good Clinical Practice.

### Inclusion and exclusion criteria

We included males and females between 20 and 55 years old who fulfilled the DSM-IV criteria for AD. To be included, participants had to report > 50% heavy drinking days within the last 90 days, and be abstinent for a minimum of four days and a maximum of 14 days before inclusion day. Individuals could not participate in the study if they were currently using any psychoactive medication, fulfilled criteria for any other substance use disorder (except nicotine) or major psychiatric disorder, or had severe somatic illness. The full inclusion and exclusion criteria are described in the supplementary information (SI).

### Study design

The study had a randomized, double-blind, placebo-controlled design. Included participants were randomized to receive either OSU or identical placebo tablets for 14 days. The patients were instructed to take the study medication twice daily (8 a.m. and 6 p.m.) according to the following dosing regimen: days 1–5, 10 mg × 2; days 6–10, 15 mg × 2; days 11–14, 30 mg × 2. The dosing regimen was adapted from previous clinical studies (e.g., Johansson et al. 2012) with the aim of minimizing the risk of patients experiencing intolerable side effects, since the study physician could decrease dosing in order to achieve a maximum tolerated dose for each participant. Medication was dispensed in three boxes for the different dosing regimens. Patients were instructed to refrain from drinking alcohol during the treatment period, but drinking during the study was not ground for study discontinuation. Importantly, each patient was instructed that they could not drink any alcohol the day before the final test day because this could bias the cognitive test session. On the test day, patients arrived at the clinic in the morning and ingested the morning dose of the study medication under supervision of a research staff member before neuropsychological testing. The participants were instructed that they could use nicotine and caffeine before arrival, but not during the cognitive testing. Further details regarding the study design, procedures, and medication have been described previously (Khemiri et al. 2015).

### Assessment instruments

Psychiatric evaluation was performed by a study M.D. using the Structured Clinical Interview for DSM-IV (American Psychiatric Association 2000) to confirm a current diagnosis of AD and exclude other severe psychiatric disorders. Alcohol consumption, craving, and mood were quantified using Time Line Follow Back interview (Sobell and Sobell 1992), Penn Alcohol Craving Scale (Flannery et al. 1999), and the Montgomery-Åsberg Depression Self-Rating Scale (MADRS-S) (Svanborg and Asberg 2001). At the end of test day, patients were asked whether they experience any “high” or “rush” from their medication (“yes” or “no”) and whether they thought they had been given active medication or placebo.

### Cognitive tests

All neuropsychological tasks were administered at baseline before first dose of assigned medication, and again on the test day after 14 days of OSU6162/placebo treatment, with the exception of the divergent thinking task and cognitive flexibility task which were only administered on the test day because of anticipated practice effects. The sequence of testing of the neuropsychological assessments was identical at both baseline and follow-up (see SI for test order). The cognitive tasks from the Cambridge Neuropsychological Test Automated Battery (CANTAB®) were administered on a tablet PC with touch screen (MOTION J3500-i7B) and press pad provided by Cambridge Cognition Ltd. For a full description of the different cognitive tests and outcomes, see original references at www.camcog.com.

#### Executive function and impulsivity

The Stop Signal Task (SST) is a test of response inhibition, i.e., the capacity to inhibit a prepotent response (Logan et al. 1984). Main outcomes of the SST were the stop signal reaction time (SSRT), median reaction time of go trials, and proportion of successful stops. Intra-Extra Dimensional Set Shift (IED) is a task of cognitive flexibility involving rule acquisition and adaptation of behavior. Main IED outcomes were numbers of extra-dimensional (ED) stage errors, pre-ED errors, stages completed, and total response latency. The Cambridge Gambling Task (CGT) is a task of decision-making and risk-taking (Rogers et al. 1999). The main outcomes of the CGT were overall proportion bet, risk-taking, delay aversion, and deliberation time. The Stockings of Cambridge (SOC) measures capacity for future planning and problem-solving, and is a development of the Tower of London (Shallice 1982; Owen et al. 1990). The main SOC outcomes were number of moves and problems solved in minimum moves for the most difficult problems (i.e., five-move problems).

#### Attention and psychomotor speed

The Rapid Visual Information Processing (RVP) task measures sustained attention (Coull et al. 1995), and the main outcomes were probability of hit, probability of false alarm, and mean latency. Attention Switching Task (AST) measures attention and set-shifting with main outcomes percent correct trials and reaction latency.

#### Working memory

The spatial working memory (SWM) is a task used to measure visuospatial working memory function (Owen et al. 1990). Main outcomes of the SWM were number of between-errors, within-errors, and strategy score. Verbal working memory was measured using the Digit Span task from the Wechsler Adult Intelligence Scale-IV (Swedish version, 2010, Pearson assessment). Main outcomes were number of correctly repeated digit sequences (total, backward, and forward).

#### Emotion

The Emotion Recognition Task (ERT) measures capacity to identify emotions from facial expressions, with main outcomes percentage correct responses and mean response latency.

#### Divergent thinking

Divergent thinking, operationalized as divergent thinking, was measured by using two timed subtests from the “inventiveness” test battery of the “Berliner Intelligenz Struktur Test” (BIS). The tests measured performance within the figural and verbal domains and were chosen based on them having the highest factor loadings on the total inventiveness-score. In the figural test, a simple line drawing was to be completed in various ways in order to create pictures of as many possible real objects as possible. In the verbal test, the participant was instructed to produce as many alternate uses for a given object as possible. Responses on both subtests are scored according to the number of produced semantic categories (i.e., semantic flexibility, not raw fluency). The test instructions provide predefined categories for a wide range of responses. Flexibility scores from each subtest were transformed into *Z*-scores, which were subsequently used as measures of divergent thinking.

### Statistical analysis

Sociodemographic and clinical baseline outcomes were compared between treatment groups using Student’s *t* test and the *χ*^2^ test of association for continuous and categorical data, respectively. Neuropsychological task outcomes were analyzed using a mixed-design analysis of variance (ANOVA) with time (pre and post treatment) as the within-subject factor and treatment (OSU and placebo) as the between-subject factor. Significant interactions were analyzed further using a repeated measures ANOVA with time as within-subject factor for each treatment group. For tasks only administered on test day, a one-way ANOVA with treatment as between-subject factor was performed. Outliers were detected by visual inspection of boxplots (defined as greater than 3 interquartile range from either quartile), and the main analyses were repeated after removing potential outliers. Exclusion of outliers did not affect statistical significance or overall interpretation of the analyses. Given the previous finding of greater treatment effect in high-impulsive subjects (based on the median split of the SSRT as baseline (Khemiri et al. 2015), we performed similar exploratory post hoc analyses to investigate whether baseline impulsivity moderated the effect on SST outcomes, as well as any other cognitive outcomes where we found a significant treatment effect in the main analyses. Effect sizes for between-group differences on test day were reported as Cohen’s *d*, i.e., mean difference divided by the pooled standard deviation (Cohen 1988).

As in previous studies employing the CANTAB® with similar hypotheses (e.g., Kehagia et al. 2014), we chose not to correct for multiple comparisons. Since this is an exploratory study of a novel drug used for the first time in this patient population, it is of great importance to detect potential drug-induced changes in cognitive function in any direction. Therefore, the risk of a type 2 errors was considered more hazardous than potential type 1 errors in this specific study setting. Thus, the alpha level was set to 0.05, two-tailed, uncorrected. All statistical analyses were done using IBM SPSS statistics (version 21.0, SPSS Inc., Chicago, Illinois).

## Results

### Study participants

Study recruitment started in September 2012 and was completed in December 2013. There were no significant differences between treatment groups at baseline regarding sociodemographic variables, alcohol consumption, dependence severity, craving, or mood (Table 1). Of the 56 included and randomized patients, one in the placebo group dropped out immediately after inclusion, due to a severe relapse. Furthermore, six individuals were excluded from the final test day when cognitive testing was to be performed, either due a positive result on the alcohol breathalyzer, or reporting of alcohol consumption the day before (*n* = 3), or a positive on the urine dip test for illicit substances (*n* = 3). Thus, the final analysis included the 49 patients who completed the entire study. One patient in each treatment group responded yes to the question of subjective “high”/“rush” when taking the medication (*χ*^2^ (1) = 0.0; *p* = 1.0), and there was no statistically significant difference between treatment groups regarding their guessed treatment assignment (*χ*^2^ (1) = 0.42; *p* = 0.52).

**Table 1:** Clinical and sociodemographic characteristics of all randomized participants. There were no significant differences between the treatment groups for any of the variables. Categorical variables are presented as percentages and continuous variables as mean (standard deviation)

	OSU6162 (*n* = 28)	Placebo (*n* = 28)	Significance
Females	50%	43%	N.S.
Age	47.3 (6.5)	45.3 (7.7)	N.S.
Education years	13.3 (2.5)	14.1 (2.8)	N.S.
Married/partner	54%	54%	N.S.
Full time employment	78.6%	71.4%	N.S.
Part time employment	7.1%	17.9%	N.S.
Unemployed	14.3%	7.1%	N.S.
Sick leave/retired	0%	3.6%	N.S.
MADRS-S score	9.2 (6.8)	7.9 (6.7)	N.S.
PACS craving score	11.1 (6.5)	10.4 (6.0)	N.S.
OCDS score	26.4 (7.4)	23.8 (5.3)	N.S.
Nicotine use daily (%)	68%	64%	N.S.
Number of DSM-IV criteria for alcohol dependence	5.2 (1.1)	5.1 (1.4)	N.S.
Percentage heavy drinking last 90 days	73%	68%	N.S.
Drinks per day last 90 days	5.8 (2.2)	5.7 (2.4)	N.S.

*MADRS-S*, Montgomery-Åsberg Depression Self-Rating Scale; *PACS*, Penn Alcohol Craving Scale; *OCDS*, Obsessive Compulsive Drinking Scale; *N.S.*, no statistically significant difference *p* > 0.05

### Cognitive testing

Baseline and test day scores on all neuropsychological test outcomes in the OSU and placebo group are presented in Table 2. There were no significant differences between treatment groups at baseline for any of the neuropsychological test outcomes (*p* > 0.05). In all analyses, the statistical significance remained unchanged even after removing individual outliers, and therefore we chose to include all participants in all analyses reported in the main manuscript.

**Table 2:** Behavioral outcomes on tasks of cognitive function in alcohol-dependent patients before and after 14 days treatment with OSU6162 or placebo. Effect sizes for between-group differences at test day are reported as Cohen’s *d*. Values are presented as mean (standard deviation) or fractions

	OSU6162		Placebo		Sig. baseline	Sig. test day	Effect size
	Baseline	Test day	Baseline	Test day			
Stop Signal Task							
SSRT	209.1 (51.5)	189.5 (62.8)	212.2 (70.3)	189.2 (52.8)	*p* = 0.863	*p* = 0.840	*d =* 0.01
Median go RT	489.7 (174.5)	448.3 (173.5)	421.9 (131.0)	388.1 (117.4)	*p* = 0.130	*p* = 0.780	*d =* 0.41
Proportion successful stops	0.52 (0.12)	0.53 (0.10)	0.49 (0.10)	0.49 (0.08)	*p* = 0.261	*p* = 0.707	*d =* 0.44
Intra-Extra Dimensional Set Shift							
ED stage errors		10.8 (11.7)		8.2 (9.2)		*p* = 0.433	*d =* 0.25
Pre-ED errors		6.7 (2.8)		5.6 (2.1)		*p* = 0.148	*d =* 0.44
Stages completed		8.5 (0.9)		8.8 (0.6)		*p* = 0.280	*d =* 0.39
Total response latency		156,699 (36,836)		156,861 (37,339)		*p* = 0.989	*d =* 0.00
Cambridge Gambling Task							
Overall proportion bet	0.57 (0.1)	0.59 (0.1)	0.51 (0.1)	0.55 (0.1)	*p* = 0.072	*p* = 0.537	*d =* 0.40
Deliberation time	2254.8 (630.7)	1847.6 (464.1)	2257.7 (630.3)	1855.0 (436.5)	*p* = 0.998	*p* = 0.970	*d =* 0.02
Risk adjustment	1.5 (0.6)	1.5 (0.6)	1.7 (0.9)	1.7 (0.9)	*p* = 0.469	*p* = 0.686	*d =* 0.26
Risk-taking	0.61 (0.1)	0.64 (0.1)	0.55 (0.1)	0.60 (0.1)	*p* = 0.081	*p* = 0.511	*d =* 0.40
Stockings of Cambridge							
Problems solved in minimum moves (five-move problems)	2.2 (1.0)	3.0 (1.0)	2.4 (1.0)	2.4 (1.2)	*p* = 0.473	*p* = 0.012	*d =* 0.54
Mean moves (five-move problems)	7.0 (1.4)	5.9 (1.2)	6.5 (1.1)	6.2 (1.1)	*p* = 0.193	*p* = 0.120	*d =* 0.26
RVP							
Probability of hit	0.59 (0.2)	0.65 (0.2)	0.60 (0.2)	0.73 (0.2)	*p* = 0.852	*p* = 0.400	*d =* 0.40
Probability of false alarm	0.0046 (0.006)	0.0054 (0.006)	0.0044 (0.004)	0.0031 (0.004)	*p* = 0875	*p* = 0.214	*d =* 0.45
Mean latency	426.8 (90)	399.7 (95.8)	420.0 (81)	393.8 (52.2)	*p* = 0.783	*p* = 0.961	*d =* 0.08
AST							
Percentage correct trials	94.7 (5.8)	96.6 (2.1)	93.2 (7.0)	95.5 (8.3)	*p* = 0.415	*p* = 0.794	*d =* 0.18
Mean latency	679.1 (174)	600.0 (165)	682.8 (194)	583.9 (152)	*p* = 0.944	*p* = 0.448	*d =* 0.10
Digit span							
Total score	16.5 (3.6)	17.4 (3.1)	15.6 (3.5)	16.6 (3.9)	*p* = 0.397	*p* = 0.877	*d =* 0.23
Backward score	7.0 (2.3)	7.3 (1.8)	6.6 (2.3)	7.3 (2.5)	*p* = 0.541	*p* = 0.478	*d =* 0.00
Forward score	9.5 (2.1)	10.0 (1.9)	9.1 (1.7)	9.4 (1.9)	*p* = 0.394	*p* = 0.631	*d =* 0.32
SWM							
Between-errors	19.4 (15)	16.9 (13)	19.0 (13)	20.6 (18)	*p* = 0.909	*p* = 0.369	*d =* 0.24
Within-errors	1.3 (2)	0.8 (1)	2.1 (4)	1.0 (2)	*p* = 0.420	*p* = 0.586	*d =* 0.13
Strategy score	29.4 (8.6)	30.3 (6.3)	28.9 (8.0)	29.7 (8.7)	*p* = 0.823	*p* = 0.9937	*d =* 0.08
ERT							
Percentage correct trials	63.3 (9.2)	66.1 (10.4)	63.3 (10.1)	68.0 (10.0)	*p* = 0.996	*p* = 0.187	*d =* 0.19
Mean latency	1840.3 (478)	1425.3 (406)	1931.5 (725)	1689.0 (738)	*p* = 0.607	*p* = 0.013	*d =* 0.44
Divergent Thinking Task							
Verbal score		5.7 (2.1)		3.9 (1.6)		*p* = 0.003	*d =* 0.96
Figurative score		5.2 (2.7)		5.0 (2.3)		*p* = 0.862	*d =* 0.08

*Sig. baseline*, *p* value of *t* test comparing outcome at baseline; *Sig. test day*, *p* value of treatment × time interaction term in the mixed ANOVA analysis for tasks administered at baseline and test day; *SSRT*, stop signal reaction time; *RT*, reaction time; *ED*, extra-dimensional; *RV*, rapid visual processing; *AST*, Attention Switching Task; *SWM*, spatial working memory; *ERT*, Emotion Recognition Task

#### Executive function and impulsivity

For the SST main outcome SSRT, there was a main effect of time (*F*_(1,47)_ = 6.7; *p* = 0.013), indicating a reduction in overall SSRT from baseline to test day (baseline 211 ms; test day 189 ms). However, there was no significant time × treatment interaction (*F*_(1,47)_ = 0.0; *p* = 0.840), or main effect of treatment (*F*_(1,47)_ = 0.0; *p* = 0.926). For all other SST outcomes, there were no significant main effects of treatment, nor treatment × time interactions (see SI for full analysis).

For the IED task (which was administered only on the test day), there were no statistically significant differences between OSU and placebo for any of the outcomes (see SI for full analysis).

In the CGT, there were no significant main effects of treatment or treatment × time interactions on any of the outcomes (see SI for full analysis). There were however significant main effects of time on several outcomes including overall proportion bet (*F*_(1,44)_ = 5.2; *p* = 0.027), deliberation time (*F*_(1,44)_ = 48.2; *p* < 0.001), and risk-taking (*F*_(1,44)_ = 5.9; *p* = 0.019). These effects indicated that the participants in general exhibited higher bets (baseline 54%; test day 57%), faster responses (baseline 1851 ms; test day 2256 ms), and increased risk-taking (baseline 58%; test day 62%) at test day compared to baseline regardless of treatment.

**Fig. 1:**
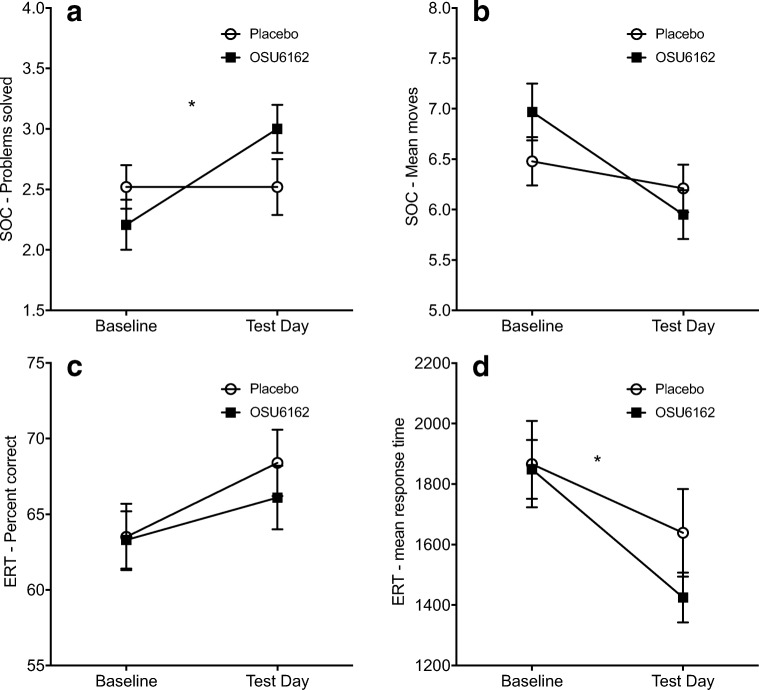
Effect of OSU6162 (OSU) compared to placebo on the Stockings Cambridge (SOC) and Emotion Recognition Task (ERT) at baseline and test day. The OSU group solved significantly more number of problems (**a**) but the treatment effect on number of moves did not reach statistical significance (**b**) in the SOC task. For the ERT, no statistically significant difference between treatment groups was found for percentage correctly identified emotion (**c**), but the OSU group had a significantly greater reduction in response time compared to the placebo group (**d**). Values are presented as mean ± s.e.m.; *p < 0.05 compared to corresponding placebo

For the SOC problems solved in minimum moves (Five-move problems), there was a significant main effect of time (*F*_(1,46)_ = 6.9; *p* = 0.012), no main effect of treatment (*F*_(1,46)_ = 0.5; *p* = 0.480) but a significant treatment × time interaction (*F*_(1,46)_ = 6.9; *p* = 0.012). Post hoc analyses showed that the OSU group solved more five-move problems on test day compared to the placebo group (*F*_(1,47)_ = 4.3; *p* = 0.043). Furthermore, only the OSU group solved significantly more problems at test day compared to baseline (*F*_(1,23)_ = 13.3; *p* = 0.001) while the placebo group exhibited no improvement between the two test sessions (*F*_(1,23)_ = 0.00; *p* = 1.00; Fig. 1a). For mean number of moves (five-move problems), there was a significant main effect of time (*F*_(1,45)_ = 7.5; *p* = 0.009), indicating a general reduction over time (baseline 6.7 moves; test day 6.1 moves) but no significant main effect of treatment (*F*_(1,45)_ = 0.2; *p* = 0.664) or treatment × time interaction (*F*_(1,45)_ = 2.5; *p* = 0.120; Fig. 1b).

#### Attention and psychomotor speed

For RVP probability of hit, there was a main effect of time (*F*_(1,46)_ = 18.6; *p* < 0.001), indicating a general improvement from baseline (59%) to test day (69%), but no main effect of treatment (*F*_(1,46)_ = 0.7; *p* = 0.400) or treatment × time interaction (*F*_(1,46)_ = 2.7; *p* = 0.106). Similar effects were found on mean latency, while no significant main effects or interactions were found on probability of false alarm (see SI for full analysis).

For the AST outcomes, there were significant main effects of time on percent correct trials (*F*_(1,47)_ = 10.0; *p* = 0.003) and mean latency (*F*_(1,47)_ = 47.2; *p* < 0.001) indicating a general improvement from baseline (correct trials 2.1% improvement; latency 89 ms decrease). No significant main effects of treatment or treatment × time interactions were found (see SI for full analysis).

#### Working memory

There were no significant main effects of time or treatment, nor any time × treatment interactions for any of the SWM task outcomes (see SI for full analysis). In the Digit span total score, there was a significant main effect of time (*F*_(1,47)_ = 5.5; *p* = 0.024) indicating a general improvement across both groups from baseline (16.1 points) to test day (17.0 points), but no significant effect of treatment (*F*_(1,47)_ = 0.7; *p* = 0.393) or treatment × time interaction (*F*_(1,47)_ = 0.0; *p* = 0.877). No significant main effects or interactions were found for forward or backward score (see SI for full analysis).

#### Emotion

In the ERT percent correct, there was a significant main effect of time (*F*_(1,47)_ = 27.9; *p* < 0.001) indicating a general improvement across groups (baseline 63%; test day 67%). However, there was no significant main effect of treatment (*F*_(1,47)_ = 0.1; *p* = 0.731; Fig. 1c) or treatment × time interaction (*F*_(1,47)_ = 1.8; *p* = 0.187). For mean latency, there was no significant main effect of treatment (*F*_(1,47)_ = 1.1; *p* = 0.303) but a significant effect of time (*F*_(1,47)_ = 98.0; *p* < 0.001) qualified by a significant treatment × time interaction (*F*_(1,47)_ = 6.7; *p* = 0.013). At test day, there was no significant difference between treatment groups (*F*_(1,47)_ = 2.4; *p* = 0.130) but the OSU group exhibited a statistically significant reduction in mean latency (mean difference − 416 ms; *F*_(1,23)_ = 91.5; *p* < 0.001), which was greater than the significant reduction in the placebo group (mean difference − 242.5 ms; *F*_(1,24)_ = 23.5; *p* < 0.001; Fig. 1d).

#### Divergent thinking

**Fig. 2:**
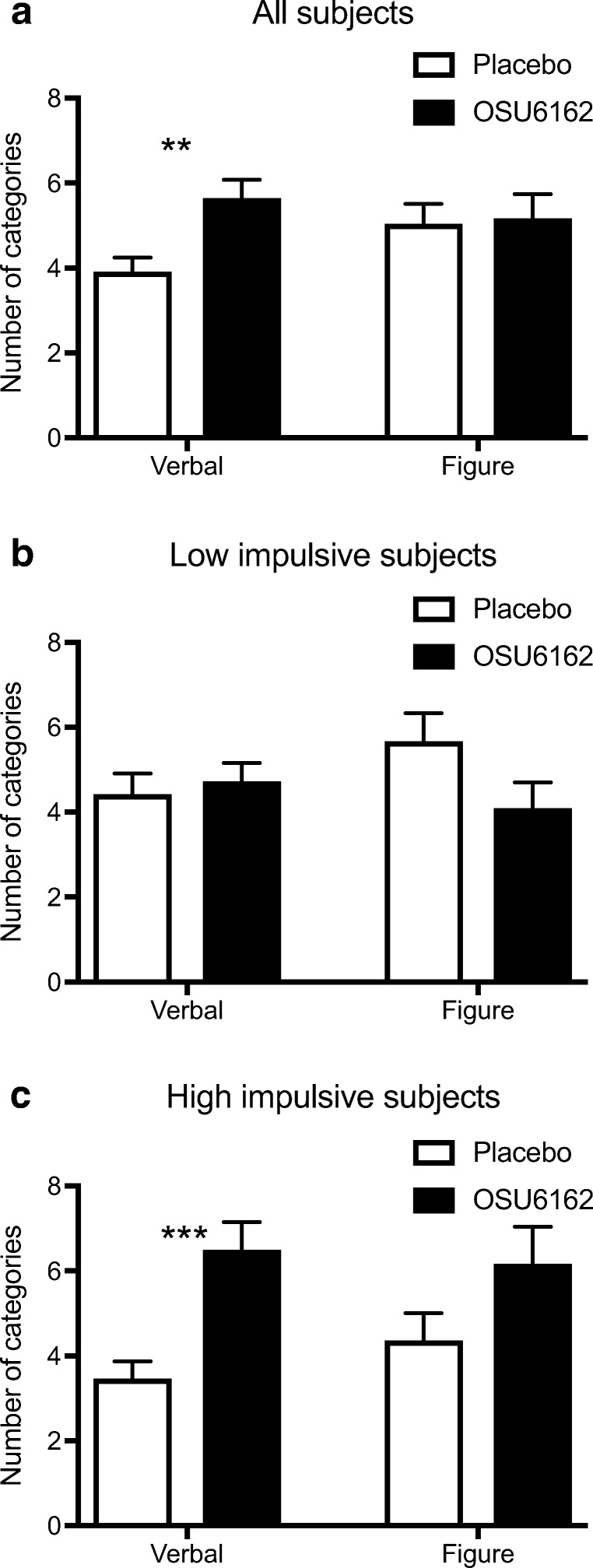
Effect of OSU6162 (OSU) compared to placebo on the Berliner Intelligenz Struktur Test of divergent thinking at test day. The OSU group exhibited greater verbal divergent thinking compared to placebo but no difference was found for the figurative outcome (**a**). In an exploratory analysis, the participants were divided into low or high impulsive based on their baseline performance on the Stop Signal Task. There were no treatment effect in the low-impulsive patients (**b**) but OSU had even greater effect on verbal divergent thinking in the high-impulsive patients (**c**). Values are presented as mean ± s.e.m.; *p < 0.05 compared to corresponding placebo

The OSU group produced a significantly greater number of semantic categories compared to the placebo group on the verbal divergent thinking task (*F*_(1,44)_ = 10.1; *p* = 0.003), but not on the figural task (*F*_(1,44)_ = 0.0; *p* = 0.862; Fig. 2a).

#### Exploratory analyses: moderating effect of baseline SSRT score

There was an even distribution of high- and low-impulsive patients within each treatment group (OSU 50% high impulsive; placebo 48% high impulsive; *χ*^2^ (1) = 0.20; *p* = 0.889). In the low-impulsive patients (lowest baseline SSRT median split), there was no main effect of time, treatment, or treatment × time interaction for the SSRT outcome. The high-impulsive group (highest baseline SSRT) however had a significant main effect of time for SSRT but no main effect of treatment or treatment × time interaction (see SI for full report of statistics).

**Fig. 3:**
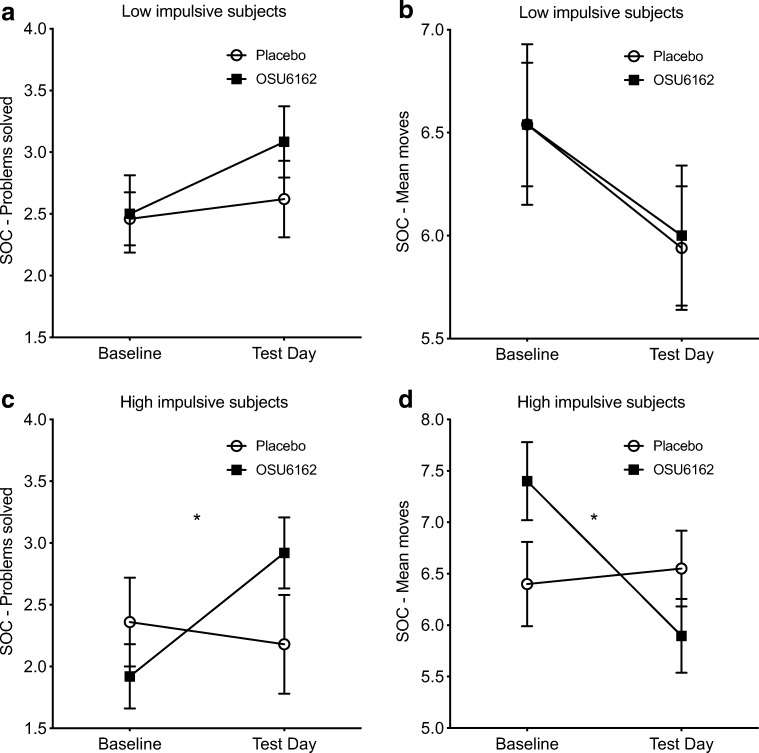
Exploratory analysis of treatment effect of OSU6162 (OSU) compared to placebo on the Stockings Cambridge (SOC) in low- and high-impulsive patients (based on baseline performance on the Stop Signal Task). In the low-impulsive patients, there were no statistically significant differences between treatment groups for number of problems solved (**a**) or performed moves (**b**). In the high-impulsive however, the OSU group improved significantly compared to placebo for both number of problems solved (**c**) and number of moves (**d**). Values are presented as mean ± s.e.m.; *p < 0.05 compared to corresponding placebo

In the low-impulsive patients, there was no main effect of time, treatment, or treatment × time interactions for the SOC outcomes (see SI for full analysis; Fig. 3a, b). In the high-impulsive group however, no significant main effect of time or treatment was found for any of the outcomes (see SI), but for SOC number of problems solved, there was a significant treatment × time interaction (*F*_(1,21)_ = 6.1; *p* = 0.022). At test day in these high-impulsive patients, there was a trend toward more problems solved in the OSU group (*F*_(1,22)_ = 3.4; *p* = 0.08) and the OSU group improved significantly at test day compared to baseline (*F*_(1,11)_ = 11.0; *p* = 0.007) while no such improvement was found in the placebo group (*F*_(1,10)_ = 0.2; *p* = 0.640; Fig. 3c). Furthermore, for the SOC mean moves on five-move problems in the high-impulsive subgroup, there was a significant treatment × time interaction (*F*_(1,20)_ = 4.7; *p* = 0.042). Post hoc analyses showed that only the OSU group improved significantly compared to baseline (*F*_(1,11)_ = 7.2; *p* = 0.021)—while no such effect was found in the placebo group (*F*_(1,9)_ = 0.1; *p* = 0.767; Fig. 3d).

For the tests of divergent thinking in the low-impulsive group, there was no statistically significant difference between the treatment groups in either verbal (*F*_(1,21)_ = 0.2; *p* = 0.645) or figural divergent thinking (*F*_(1,21)_ = 3.0; *p* = 0.098; Fig. 2b). In the high-impulsive patients however, the OSU group scored significantly higher on verbal divergent thinking than the placebo group (*F*_(1,21)_ = 16.1; *p* < 0.001). No difference in figural divergent thinking was found between the groups (*F*_(1,21)_ = 2.7; *p* = 0.117; Fig. 2c).

For ERT response latency in contrast, no significant treatment × time interaction was found in the high-impulsive patients (*F*_(1,22)_ = 0.3; *p* = 0.576), but a treatment effect was found in the low-impulsive subjects (*F*_(1,23)_ = 9.3; *p* = 0.006; see SI for full analysis).

## Discussion

In the present randomized placebo-controlled laboratory study, we found that 14 days of treatment with the novel monoamine stabilizer OSU did not cause any short-term negative effects on any of the assessed domains of cognitive function in AD patients. Our results in fact provide preliminary evidence that OSU treatment may exert positive effects on specific cognitive functions, including future planning ability, verbal divergent thinking, and emotional recognition speed.

The potential negative impact of pharmacological agents on cognition is an important and known clinical problem (Campbell and Boustani 2015), and it is well established that drug-induced hyperactivation of the dopamine system, e.g., by L-DOPA, is associated with impaired impulse control (O’Sullivan et al. 2009; Corvol et al. 2018). In the current study, we found no negative effect of OSU treatment on cognitive function, including tasks related to impulsivity and executive function, after 14 days treatment. Early onset of side effects is a common reason for non-adherence to pharmacological therapy, e.g., in the case of antidepressants (Fortney et al. 2011). The lack of cognitive side effects found in the current short-term study, together with the generally mild side effect profile reported in previous studies of AD (Khemiri et al. 2015) and other patient populations (Rodríguez et al. 2004; Johansson et al. 2012; Kloberg et al. 2014; Nilsson et al. 2018), therefore support the clinical feasibility of OSU and its continued evaluation in clinical populations.

The current findings suggest a positive effect of OSU treatment on future planning capability. OSU-treated patients compared to the placebo group managed to solve significantly more of the most difficult problems in the SOC task. There was however no statistically significant difference in the number of performed moves, even though the direction of the association was indicative of greater decrease in the OSU group compared to placebo. Studies of the antipsychotic dopamine D2/D3 receptor antagonist sulpiride have shown conflicting results on similar tasks of future planning. A dose of 400 mg sulpiride has been found to both impair (Mehta et al. 1999) and improve (Mehta et al. 2003) future planning ability. However, the most recent study found that a larger dose of 800 mg induced impairments in SWM task performance and in performance on the One-Touch SOC task (Naef et al. 2017), which is similar to the SOC task used in the current study. In contrast, OSU treatment in the present study had no negative effect on SWM task performance while improving future planning ability assessed by the SOC task. Our finding could be explained by the fact that OSU has a proposed pharmacological mechanism of action suggested to be different from traditional antipsychotic medications (Sonesson et al. 1994; Burstein et al. 2011; Carlsson et al. 2011), or perhaps through its partial agonist action at the 5HT2A receptor (Burstein et al. 2011; Carlsson et al. 2011). Elevated impulsivity and poorer response inhibition are known to be related to a stronger craving reaction in AD patients (Papachristou et al. 2013), and our finding of an improved ability of future planning in the OSU group could explain earlier results of a reduced craving response in patients with AD (Khemiri et al. 2015). Taken together, these preliminary findings suggest that one mechanism through which OSU can reduce craving may be through modulation of future planning capacity, suggesting a value in including such assessments in future trials investigating the effect of OSU in AD patients.

Divergent thinking, which indicates creative potential, has previously been found associated with the dopamine system and specifically reduced D2 receptor binding in the thalamus, in healthy individuals (de Manzano et al. 2010). Divergent thinking is dependent on both generation and selection of responses, i.e., both imagery/free association and top-down executive control, as well as switching between brain networks associated with these functions (Pinho et al. 2016). In the current study, we found that OSU-treated patients performed better than the placebo group on a verbal subtask, which measures semantic flexibility and evaluates the participants’ ability to produce as many alternate uses as possible, for a certain object. This improvement, which would involve an improvement in semantic retrieval and/or top-down control of free association, might beyond the general benefits of divergent thinking and control of thought processes be particularly important also for relapse prevention skills which includes identifying different coping strategies when experiencing craving for alcohol (Witkiewitz and Marlatt 2004). Finally, we also found an effect of OSU on response times on the ERT task, indicating that OSU-treated participants had a greater reduction in response time compared to the placebo group. In a recent study, it was found that poor emotion recognition ability predicted worse treatment outcome in AD patients (Rupp et al. 2017), suggesting that assessment of emotional recognition has clinical importance, and that improvement of this cognitive domain could represent a potential treatment target. The clinical importance of the observed reduction in emotional recognition response time is not known, but may reflect a faster and thereby improved understanding of other peoples’ emotions. This could putatively reduce risk of interpersonal conflict and stress, which in turn are known to increase craving and risk of relapse (Sinha 2007). However, we emphasize that our findings regarding the positive treatment effect of OSU on all the aforementioned cognitive outcomes are tentative, and further studies of the effects of OSU on cognition are needed to confirm these preliminary findings.

As mentioned earlier, in a preclinical study, OSU was found to improve motor impulsivity on the 5-choice serial task in alcohol-naïve and long-term drinking rats (Fredriksson et al. 2018). In the current study, however, we failed to translate the preclinical finding on motor impulsivity, as we found no significant time-by-treatment interaction on the equivalent human cognitive task, i.e., the SST. We also did not detect any baseline performance-dependent effect of treatment on SSRT that has previously been reported in AD patients for other dopaminergic agents such as modafinil (Schmaal et al. 2013). At present, the reason for the discrepancy between the preclinical and clinical findings is not clear. One possibility is that alcohol intake during the study hampered any potential improvements in motor impulsivity, given the negative acute effects of alcohol intake on response inhibition (Schweizer et al. 2006). Another possible explanation is that our treatment period of only 14 days may have been too brief to detect the putative effect on motor impulsivity in individuals with a current diagnosis of AD. Finally, given our repeated measures design, and significant main effects of time indicating an overall improvement in all patients, it is possible that potential treatment effects were usurped by natural recovery and/or practice effects.

We have previously reported that high baseline impulsivity defined by SSRT median split predicts greater treatment response to OSU (Khemiri et al. 2015). In the current analysis however, no moderating effect of baseline SSRT was found on the treatment effect on SST performance. For two of the neuropsychological task outcomes significantly improved by OSU in the main analysis (i.e., SOC and verbal divergent thinking task), the OSU treatment effect was indeed only significant in patients with high impulsivity at baseline. Interestingly, similar findings of a moderating effect of baseline impulsivity on treatment outcome have been found for other dopaminergic pharmacological agents such as modafinil (Schmaal et al. 2013; Joos et al. 2013b) and aripiprazole (Voronin et al. 2008; Anton et al. 2017) in AD patients. Although the underlying neurobiological mechanism is currently not known, several lines of research indicate an association between dopaminergic neurotransmission, impulsivity, and substance use. For instance, rats with high trait impulsivity exhibit lower D2 receptor availability as well as elevated cocaine intake (Dalley et al. 2007), and in a rat model of chronic intermittent ethanol exposure, alcohol caused executive function deficits coupled with disruption of D2/D4 DA receptor signalling in the medial prefrontal cortex (Trantham-Davidson et al. 2014). Furthermore, ventral striatal brain activity, putatively mediated in part by dopaminergic signalling, was associated with self-rated impulsivity during a reward anticipation task in AD patients (Beck et al. 2009). It has also been suggested that genes related to dopaminergic function, e.g., the dopamine D2 receptor, are associated with AD (Munafò et al. 2007), impulsivity (Taylor et al. 2017; Kim et al. 2018), and a reduction in D2 receptor levels (Buckholtz et al. 2010). Collectively, these findings support the proposition that high baseline impulsivity could be a marker of altered dopaminergic neurotransmission, which in turn drives substance use (Kozak et al. 2018). This mechanism could explain why dopaminergic acting agents can have differential action on this subgroup of AD patients with elevated impulsivity. Whether specific genetic markers involved in DA neurotransmission can predict cognitive profiles as well as treatment response to dopaminergic pharmacological agents such as OSU is an interesting question for future research.

This study has several important limitations which need to be addressed. Firstly, we performed multiple statistical tests without correcting the significance threshold for multiple comparisons. In agreement with previous studies (e.g., Lees et al. 2017), we argue that it is justified to perform such exploratory uncorrected analyses in the early stages of investigating pharmacological agents, as long as results are interpreted cautiously. Since this is a pioneering study of a novel drug used for the first time in this patient population, it is of interest to explore all potential drug-induced changes in cognitive function in any direction. It is therefore also interesting to note that all statistically significant cognitive outcomes were in the same direction, i.e., they indicated a change toward improvement rather than deterioration in cognition. Secondly, the sample size was limited (*n* = 56), allowing the detection of only moderate to large effect sizes. It is possible that larger samples are needed to detect additional effects on cognitive function. Thirdly, we utilized a test-retest design which may induce practice effects for the different cognitive tasks, thereby masking potential treatment effects. Fourth, the study sample comprised AD patients with relatively good social function (majority full time employed) and without severe psychiatric co-morbidity. Our findings might therefore not generalize to a more severe clinical AD patient population. Finally, 69% of the participants consumed alcohol during the study. Even though there were no difference between the groups regarding drinking (Khemiri et al. 2015), it is possible that potential treatment effects on cognition could be more accurately assessed if the study had been performed at an inpatient unit without concurrent alcohol intake.

In summary, short-term treatment with OSU had no significant negative impact on any measured cognitive function compared to placebo in AD patients. In contrast, positive effects of OSU were found on planning ability, verbal divergent thinking, and emotional recognition, which may potentially be relevant for clinical outcomes. Our results further support the potential of OSU as a clinically feasible and safe therapeutic in AD patients. Future clinical studies of OSU should also administer neuropsychological tasks as part of the assessments, in order to further clarify the effect of OSU on cognitive function.

## Electronic supplementary material

ESM 1 (DOCX 25 kb)
