# Supplementary material for: Effects of the monoamine stabilizer (-)OSU6162 on cognitive function in alcohol dependence
Source: Psychopharmacology (Berl). 2019 Oct 18;237(1):69–82. doi: 10.1007/s00213-019-05345-6 (PMC6952337; doi:10.1007/s00213-019-05345-6)
Supplement: Supplementary file 1 — (DOCX 25 kb) [file 213_2019_5345_MOESM1_ESM.docx]

S2. Materials and Methods

Inclusion criteria:

- Male or a non-pregnant/non-nursing female between 20 and 55 years of age
- Minimum nine years of education
- Fulfills the criteria for alcohol dependence according to DSM-IV
- Self-report of minimum 50 percent heavy drinking days (5 and 4 daily standard drinks or more for males and females, respectively) during a 60 day period, within the last 90 days before screening
- Minimum 4 and maximum 14 abstinent days before day of inclusion
- Be willing to give informed consent and comply with study procedures.

Exclusion criteria:

- Current use of any psychoactive medication
- Fulfills DSM-IV criteria for the following axis 1 disorders: Schizophrenia, bipolar disorder, major depression or any other substance dependence disorder (excluding nicotine)
- Clinically significant alcohol withdrawal on day of inclusion
- Previous withdrawal-induced delirium tremens or seizures
- Presence of any serious somatic disorder e.g. liver cirrhosis
- Presence of any previous heart disease of clinically significant ECG abnormality e.g. arrhythmias, bundle branch block or QT interval or T wave alterations
- Intermediate or high probability of long QT syndrome (LQTS) defined by the LQTS diagnostic criteria
- Use of any illegal narcotic drugs the last 30 days before screening
- Traces of any illegal narcotic substance i.e. amphetamine, cocaine, THC or opiates in urine sample on day of inclusion

Neuropsychological test order:

The sequence of testing of the neuropsychological assessments were identical at both baseline and follow-up, and the tests were presented in the following order: Digit Span - Stop Signal Task – Emotion Recognition Task – Attention Switching Task – Spatial Working Memory task – Cambridge Gambling Task – Rapid Visual Information Processing – Stockings of Cambridge. The divergent thinking task was only administered at test day and was administered before the other neuropsychological tasks.

S3. Results

SST
For the SST main outcome SSRT, there was a main effect of time (F_(1,47)_=6.7; p=0.013) indicating a reduction in overall SSRT from baseline to test day (baseline 211; test day 189). However, there was no significant time*treatment interaction (F_(1,47)_=0.041; p=0.840) or main effect of treatment (F_(1,47)_=0.01; p=0.926). The median go reaction time data was logarithmized because of positive skew. There was a main effect of time (F_(1,47)_=12.0; p=0.001) indicating a reduction in median go reaction time (baseline 455 ms; test day 418 ms), but no main effect of treatment (F_(1,47)=_2.4; p=0.125) or time*treatment interaction (F_(1,47)_=0.1; p=0.780). For proportion of successful stops there was no significant main effect of time (F_(1,47)_=0.1; p=0.817), treatment (F_(1,47)_=2.5; p=0.124) or time*treatment interaction (F_(1,47)_=0.1; p=0.707). Finally, for standard deviation of go trials, there was a main effect of time (F_(1,47)_=9.6; p=0.003) indicating a general reduction (baseline 150; test day 101). No significant main effect of treatment (F_(1,47)_ =2.0; p=0.165) or time*treatment interaction was found (F_(1,47)_ =0.2; p=0.622).

IED
For extra-dimensional errors (t_(39)_ =0.8; p=0.433), pre-extra-dimensional errors (t_(39)_ =1.5; p=0.148), total stages completed (t_(39)_ =-1.1; p=0.280) or total latency (t_(39)_ =-0.1; p=0.989).

CGT
In the CGT outcome overall proportion bet, there was a significant main effect of time (F_(1,44)_ =5.2; p=0.027), indicating a general increase over time (Mean proportion bet across groups at baseline: 0.54; Test day: 0.57). No significant main effect of treatment (F_(1,44)_ =2.9; p=0.093) or treatment*time interaction (F_(1,44)_ =0.4; p=0.537) was found however. For CGT deliberation time similarly, there was a significant main effect of time (F_(1,44)_ =48.2; p<0.001) indicating generally reduced deliberation time at test day (overall mean 2256 ms) compared to baseline (overall mean 1851 ms). There was no significant main effect of treatment (F_(1,44)_ =0.0; p=0.973) or treatment*time interaction (F_(1,44)_ =0.0; p=0.970). For risk taking, there was a significant main effect of time (F_(1,44)_ =5.9; p=0.019), indicating a general increase over time (Mean risk taking at baseline: 0.58; Test day: 0.62). No significant main effect of treatment (F_(1,44)_ =2.6; p=0.114) or treatment*time interaction (F_(1,44)_ =0.4; p=0.511) was found. For CGT risk adjustment, there was no significant main effect of time(F_(1,44)_ =0.0; p=0.956), treatment (F_(1,44)_ =1.0; p=0.314) or treatment*time interaction (F_(1,44)_ =0.2; p=0.686).

RVP
For RVP probability of hit, there was a main effect of time (F_(1,46)_ =18.6; p=0.000), indicating a general improvement from baseline (0.59) to test day (0.69), but no main effect of treatment (F_(1,46)_ =0.7; p=0.400) or treatment*time interaction (F_(1,46)_ =2.7; p=0.106). For probability of false alarm, there was no significant main effect time (F_(1,46)_ =0.1; p=0.754), treatment (F_(1,46)_ =1.0; p=0.316) or treatment*time interaction (F_(1,46)_ =1.6; p=0.214). For mean latency, there was a significant main effect of time (F_(1,46)_ =5.8; p=0.020) indicating a general reduction between baseline (423 ms) and test day (397 ms) – while no significant main effect of treatment (F_(1,46)_ =0.1; p=0.762)) or treatment*time interaction (F_(1,46)_ =0.0; p=0.961) were found.

AST
For the AST percentage correct trials, there was a significant main effect of time (F_(1,47)_ =10.0; p=0.003) indicating an overall improvement between test sessions (Baseline: 93.9 %; Test day: 96.0 %) while no significant main effect of treatment (F_(1,47)_ =0.6; p=0.427) or time*treatment interaction (F_(1,47)_ =0.1; p=0.794) were found. For mean latency, there was a significant main effect of time (F_(1,47)_ =47.2; p<0.001), indicating a general reduction from baseline (681 ms) to test day (592 ms), but there were no significant main effect of treatment (F_(1,47)_ =0.0; p=0.897) or treatment*time interaction (F_(1,47)_ =0.6; p=0.448).

SWM
For SWM between-errors, there was no significant main effect of time (F_(1,47)_ =0.0; p=0.854), treatment (F_(1,47)_ =0.2; p=0.644) or treatment*time interaction(F_(1,47)_ =0.8; p=0.369). For within-errors, there was no significant main effect of time (F_(1,47)_ =3.4; p=0.072), treatment (F_(1,47)_ =0.8; p=0.381) or treatment*time interaction (F_(1,47)_ =0.3; p=0.586). For SWM strategy score, there was no significant main effect of time (F_(1,47)_ =1.4; p=0.245), treatment (F_(1,47)_ =0.1; p=0.784) or treatment*time interaction (F_(1,47)_ =0.0; p=0.9937).

Digit Span
In the Digit span total score, there was a significant main effect of time (F_(1,47)_ =5.5; p=0.024) indicating a general improvement across both groups from baseline (16.1) to test day (17.0), but no significant effect of treatment (F_(1,47)_ =0.7; p=0.393) or treatment*time interaction (F_(1,47)_ =0.0; p=0.877). No significant main effects or interactions were found for forward or backward score (See SI for full analysis). For forward score, there were no significant main effects of time (F_(1,47)_ =2.9; p=0.097), treatment (F_(1,47)_ =0.1; p=0.743) or treatment*time interaction (F_(1,47)_ =0.5; p=0.478). Similarly for backward score, there were no significant main effects of time (F_(1,47)_ =2.9; p=0.093), treatment (F_(1,47)_ =1.4; p=0.243) or treatment*time interaction (F_(1,47)_ =0.2; p=0.631)

Post hoc analysis: Moderating effect of baseline SSRT score

In the low-impulsive subjects there was no significant main effect of time (F_(1,23)_ =0.1; p=0.711), treatment (F_(1,23)_ =0.0; p=0.902) or treatment*time interaction (F_(1,23)_ =0.00; p=0.996) for the SSRT outcome. In the high-impulsive subjects, there was a significant main effect of time (F_(1,22)_ =16.7; p=0.001) indicating general reduction in SSRT across sessions (Baseline: 255 ms; Test day: 208 ms). No significant main effect of treatment (F_(1,22)=_0.0; p=0.861) or time*treatment interaction (F_(1,22)_ =0.1; P=0.724) was found.

In the low-impulsive subjects (lowest baseline SSRT), there was no main effect of time (F_(1,23)_ =3.8; p=0.064) treatment (F_(1,23)_ =0.5; p=0.481) or treatment*time interaction (F_(1,23)_ =1.3; p=0.269) for the SOC problems solved in minimum moves (5-move problems). In the high-impulsive group however, no significant main effect of time (F_(1,21)_ =2.9; p=0.102) or treatment (F_(1,21)_ =0.1; p=0.721) was found, but there was a significant treatment*time interaction (F_(1,21)_ =6.1; p=0.022). At test day in the high-impulsive subjects, there was a trend toward more problems solved in the OSU group (F_(1,22)_ =3.4; p=0.08) and the OSU group improved significantly at test day compared to baseline (F_(1,11)_ =11.0; p=0.007) while no such improvement was found in the placebo group (F_(1,10)_ =0.2; p=0.640).

For SOC mean moves in 5-move problems in the LOW impulsive subjects, there was no main effect of time (F_(1,23)_ =4.0; p=0.059), treatment (F_(1,23)_ =0.0; p=0.936) or treatment*time interaction (F_(1,23)_ =0.0; p=0.925). In the HIGH impulsive subgroup however, there was no main effect of time (F_(1,20)_ =3.2; p=0.090), treatment (F_(1,20)_ =0.2; p=0.659) but a significant treatment*time interaction (F_(1,20)_ =4.7; p=0.042). Post hoc analyses showed that at test day, there was no significant difference between treatment groups (F_(1,20)_ =1.6; p=0.218) but only the OSU group improved significantly compared to baseline (F_(1,11)_ =7.2; p=0.021) but no such effect was found in the placebo group (F_(1,9)_ =0.1; p=0.767).

For the verbal creativity task score in the low impulsive group, there was no statistically significant difference between treatment groups (OSU: 4.7 (1.6); Placebo: 4.4 (1.7); F_(1,21)_ =0.2; p=0.645). In the high impulsive subjects however, the OSU group scored significantly higher compared to the placebo group (OSU: 6.5 (2.2); Placebo: 3.4(1.4); F_(1,21)_ =16.1; p=0.001).

For ERT response latency in contrast, the treatment effect was manifested in the low impulsive subjects only, where a significant main effect of time (F_(1,23)_ =52.5; p<0.0001), no significant main effect of treatment (F_(1,23)_ =1.3; p=0.262) but a significant treatment*time interaction was observed (F_(1,23)_ =9.3; p=0.006). Both groups had a significant reduction in response latency, but the magnitude was greater in the OSU group (Mean reduction:-490 ms; F_(1,11)_ =52.9; p<0.0001) compared to the placebo group (Mean reduction: -299 ms; F_(1,12)_ =8.9;p=0.012). In the high impulsive subjects however, there was also a significant main effect of time (F_(1,22)_ =49.5; p<0.0001), a significant main effect of treatment (F_(1,22)_ =4.7; p=0.041) but no significant treatment*time interaction (F_(1,22)_ =0.3; p=0.576). For ERT percentage correct, there were no significant treatment*interaction for neither the low-impulsive (F_(1,23)_ =0.5; p=0.489) or high-impulsive (F_(1,22)_ =1.3; p=0.273) group.
